# Genetic characterization of the indigenous Cyprus cattle breed

**DOI:** 10.1007/s11250-025-04728-6

**Published:** 2026-03-26

**Authors:** Mikaella Kyriakou, Simoni Symeou, Ouranios Tzamaloukas, Despoina Miltiadou

**Affiliations:** Department of Agricultural Sciences, Biotechnology and Food Science, Cyprus University of Technology, PO Box 50329, Lemesos, 3603 Cyprus

**Keywords:** Microsatellite analysis, Cattle, Local breed, Bos taurus indicus, Cyprus bovine zebu breed

## Abstract

The Cyprus Bovine Breed, an indigenous cattle population, faces ongoing challenges in preserving its genetic diversity due to the introduction of high yielding commercial breeds and the global economic development. This study investigates the genetic structure of the breed using 18 microsatellite markers and compares it to both commercial breeds in the island and global cattle populations. Nearly 10% of the total population, in particular 116 individuals of the Cyprus Bovine Breed, were analyzed and the findings revealed moderate to high genetic diversity (Ho = 0.691, He = 0.714), with allele richness comparable to other indigenous breeds (Na = 6.265). Notably, there is no evidence of genetic admixture with commercial cattle breeds grown in Cyprus. The analysis also highlights low inbreeding (Fis = 0.066) and genetic cohesion across regions of Cyprus. It was found that the Cyprus Bovine Breed shows genetic affinities to both Zebu and African Bos taurus cattle, reflecting a complex history of genetic exchanges likely driven by ancient trade and migration routes. These results have important implications for the conservation of the Cyprus Bovine Breed. The breed’s genetic diversity and minimal differentiation among regions suggest that it has retained considerable genetic variability despite modern challenges. To ensure its survival, sustainable breeding practices and region-specific programs are essential to maintain its genetic integrity. The results can find practical application in conservation programs of the Cyprus bovine endangered breed, as well as become the basis for future fundamental research.

## Introduction

The genetic characterization of livestock is essential for the conservation and sustainable management of animal genetic resources. Indigenous cattle breeds, such as the Cyprus cattle, are vital genetic resources for rural communities, providing nutritional, economic and socio-cultural benefits (Mapiye et al. 2020). These breeds are well adapted to harsh climates, exhibit resilience to local diseases, parasites and climate extremes, and possess unique qualities that can contribute to food security and agricultural sustainability. They are capable of thriving on poor-quality forages, making them essential for sustainable, low-input agricultural systems (Rahal et al. 2020; Mamogobo et al. 2021; Solodneva et al. 2023).

The indigenous Cyprus cattle breed, like many indigenous breeds, face the threat of extinction, according to a global inventory of animal diversity (Mathew and Mathew 2023), due to the requirements of intensive livestock farming and global economic development, leading to a progressive replacement of traditional multipurpose breeds by high-yielding breeds and more profit-oriented farming (Marsoner et al. 2018). Indeed, the number of native breeds has diminished and it is estimated that nearly 30% of native breeds worldwide are endangered (Ovaska et al. 2021). The genetic erosion and extinction of domesticated animal species is a global issue, with 1–2 breeds, including indigenous cattle, disappearing weekly (Solodneva et al. 2023).

The Cyprus Bovine breed is a unique native breed reared in Cyprus. The Cypriot bovine (Bos taurus indicus) is characterized by the hump, the relatively large dewlap, the black tuft at the tip of the tail and the whitish-gray ring that surrounds its black lips and nostrils. On the lower part of the limbs and at the base of the hooves, where the black ring is visible, there is a lighter white or yellowish coat coloration. Formerly, they were classified into two types, those of the mountainous areas or Paphos and those of the lowland areas or Mesaoria. The animals from the mountainous areas were small, with coarse hair in all shades of brown, though black-colored animals were also found. In contrast, the animals from the lowland areas had a robust body structure, fine hair and a reddish-blonde coloration with whitish coloring on the abdomen. The animals were used as a source of power for agricultural tasks (plowing, threshing) and for the transportation of people and goods. Nowadays, the animals are used for meat production which might be of exceptional quality, as the animal is resilient to the unique conditions of Cyprus and the breed primarily obtains its feed from grazing, in contrast with the main farmed breed in Cyprus, the Holstein-Friesian, which is bred inside, in an intensive farming environment. Current counts of the Cyprus bovine breed sum up to approximately 1200 animals and if this number continues to decline, inbreeding might put the future of this breed at stake (Cypriot Ministry of Agriculture 2023). Therefore, it is essential to detect the levels of existing genetic diversity and population structures, facilitating the development of national breeding strategies to preserve its unique characteristics and ensure its survival.

In recent years, significant progress in the characterization of indigenous breeds has been made in most countries (Svishcheva et al. 2020). In the Mediterranean region, Ciampolini, Mastrangelo and Goudarzi (2015) explored genomic regions related to climate resilience, using Italian and Corsican local breeds as key targets, while the main objective of the study supported by Gamarra et al. (2020) was to investigate the phylogenetic relationships of Pirenaica cattle and other breeds raised in the Basque region, aiming to identify several STR markers for parentage and traceability purposes. In Beja-Pereira et al. (2003) focused on the cattle breeds of the Iberian Peninsula, while Cymbron et al. (2005) extended this exploration of genetic diversity to 20 cattle breeds from the Mediterranean and Northern Europe. Another study by Dalvit et al. (2008), analyzed Italy’s Burlina breed in comparison to two widely used dairy cattle, Holstein-Friesian and Brown Swiss. Baladi cattle, a native breed found throughout the Mediterranean, have been highlighted by Shabtay (2015) for their adaptability and efficiency, outperforming large-framed cattle in both feedlots and Mediterranean pastures and lastly, Mamogobo et al. (2021) assessed the genetic characterization of non-descript cattle populations in South African communal areas.

The Cypriot cattle breed has received limited attention in genomic research, which impedes the development of effective conservation strategies. The only available studies including this breed were developed by Flori et al. (2019) and Papachristou et al. (2020), using nine and five animals, respectively. In one of the mentioned studies (Papachristou et al. 2020), the Cypriot cattle breed was compared with 104 international breeds using more than 46,000 single nucleotide polymorphisms (SNPs), while Flori et al. (2019) assessed the genetic diversity of 21 autochthonous cattle breeds from the Mediterranean region.

In the current study, nearly 10% of the Cyprus cattle population has been sampled for the first time to genetically characterize the indigenous Cyprus cattle breed, providing information about genetic diversity, structure and differentiation of the breed compared to both imported commercial breeds farmed in the island and to a large dataset including 25,000 animals from all over the world.

## Materials and methods

### Blood samples collection

**Fig. 1:**
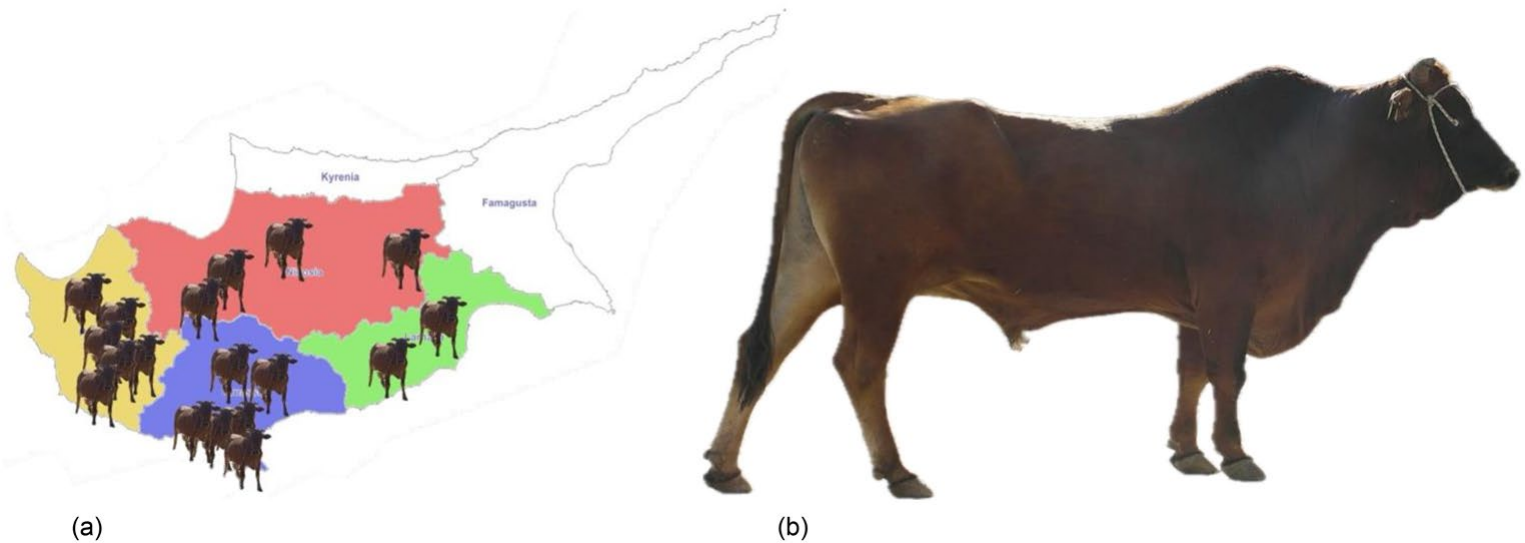
(**a**) Map of the sampled animals within the four provinces of Cyprus, blue indicating Limassol, green indicating Larnaca, red indicating Nicosia and yellow indicating Paphos, and (**b**) representative type of the Cypriot Bovine Breed cattle

Blood samples were obtained from 179 individual cattle, both male and female (Table 1), across four distinct Cyprus prefectures (Fig. 1a). Most samples (116) were from the Cypriot local bovine cattle (Fig. 1b) and the remaining (63) were from commercial cattle breeds (Black Angus, Charolais, Holstein-Friesian, and Jersey) farmed in Cyprus. Collection occurred during multiple field sampling missions in Limassol, Larnaca, Nicosia and Pafos, involving agricultural research institutions and cattle herds in Cyprus (2023). To ensure a low degree of relatedness among sampled individuals, efforts were made to select unrelated animals from different herds based on farmers’ feedback. Blood sampling followed established protocols regarding animal handling and ethics (Republic of Cyprus 1994; European Union 2010). Specifically, animals were restrained in a safe and stress-free manner, and 18 mL of whole blood was drawn from the coccygeal vein into two NH Sodium Heparin-containing collection tubes (9 ml NH Sodium Heparin, VACUETTE^®^ TUBE, Greiner Bio-One, Kremsmünster, Austria). The use of NH Sodium Heparin as an anticoagulant is a widely advised method for preserving the integrity of blood samples, without interfering with DNA extraction methods (Kotikalapudi and Patel 2015).

**Table 1:** Sampling scheme across Cypriot provinces and villages for the five cow breeds analyzed

Breed	Province	Village	Samples number
Cyprus Bovine Breed	Larnaca	Kalo Chorio	1
		Kofinou	10
	Paphos	Marathounta	12
		Pentalia	2
		Kannaviou	6
		Phaleia	15
		Peristerona & Steni	4
		Houlou	2
	Limassol	Akrotiri	24
		Episkopi	3
		Erimi	5
		Apsiou	9
		Paramali	2
		Doros	5
	Nicosia	Yeri	5
		Kalopanayiotis	11
Jersey	Nicosia		24
Holstein-Friesian	Nicosia		26
Black Angus	Nicosia		6
Charolais	Nicosia		7

### DNA extraction and quantification

Total DNA was extracted using the NucleoSpin^®^ Blood kit, following the manufacturer’s instructions, using 2 mL of blood (MACHEREY-NAGEL, Düren, Nordrhein-Westfalen, Germany). The concentration and purity of DNA were assessed using a NanoDrop 1000 spectrophotometer in the UV light range (220–350 nm) (Thermoscientific, Waltham, MA, USA). DNA integrity was further verified by 2% agarose gel electrophoresis. DNA samples were diluted with ddH_2_O to a 1–3 ng/ uL concentration and kept at -20 °C until PCR amplification.

### Multiplex PCR amplification and fragment analysis

Multiplex polymerase chain reaction (PCR) amplification was conducted using the Thermo Scientific Bovine Genotypes Panel 3.1 kit, following the manufacturer’s instructions, with minor adjustments (Thermo Fisher Scientific Inc., Waltham, MA, USA). Eighteen loci were simultaneously amplified using 5’-labeled primers (FAM TM, JOE TM, NED TM, and ROX TM). These included all 12 STR loci (BM1818, BM1824, BM2113, ETH10, ETH225, ETH3, INRA23, SPS115, TGLA122, TGLA126, TGLA227, and TGLA53) that are approved by the International Society for Animal Genetics (ISAG) for regular use in parentage testing and identification (FAO/ISAG 2004). Furthermore, six extra microsatellites (CSRM60, CSSM66, ILSTS006, MGTG4B, RM067, SPS113), which are recommended loci by the Food and Agriculture Organization of the United Nations (FAO) for genetic research of domestic animals, were co-amplified (FAO/ISAG 2004).

For PCR reactions, the mix contained 1 ng of template DNA in a 10 µL final reaction volume. Conditions for the PCR amplification were as follows: An initial denaturation step (98 °C for 60 s) followed by 30 amplification cycles (98 °C for 20 s, 60 °C for 75 s, 72 °C for 30 s). A final elongation step was lastly included (72 °C for 5 min). PCR products were kept at -10 °C until analysis. Amplification products were verified using a standard 2% agarose electrophoresis and several dilution rates (1:1, 1:5, 1:10 and 1:20 with ddH_2_O) were tested to optimize capillary electrophoresis. Optimal results were attained at a 1:10 ratio (data not shown). One µL of the dilutions was added to 8.9 µL deionized formamide and 0.1 µL of DNA size standard (GeneScan 500-LIZ, Applied Biosystems, Foster City, CA, USA), before denaturing for 5 min at 95 °C. Positive (Bovine Genotypes Control DNA001) and negative controls (H_2_O) were additionally included to exclude accidental contaminations and for amplicon harmonization across the plates. Allelic fragments were separated by capillary electrophoresis using an SeqStudio Genetic Analyzer (Applied Biosystems, Foster City, CA, USA).

### De-multiplexing and allele size calling

Allele sizes per genetic locus were determined using the SeqStudio™ suite and analyzed with the GeneMapper™ program (Thermo Fisher Scientific Inc., Waltham, MA, USA). Bin sizes were selected based on bibliography/kit user guide for each locus and according to the fluorochrome. This allowed the identification of fragments ranging from 63 to 309 base pairs (bp). Two different researchers performed allelic calling, while common template DNA controls and samples across plates allowed the harmonization of fragments’ size.

### Population structure and differentiation

The MS Excel add-in GENALEX v. 6.501 was used to curate and format microsatellite data (Peakall and Smouse 2012). For computing allelic frequencies across the examined loci, all isolates were taken into consideration. A genotype accumulation curve was created to evaluate the ability to distinguish between unique individuals. Additionally, several indices were used to measure genotypic diversity, including the Number of Alleles (Na), Effective Number of Alleles (Ae), the expected Heterozygosity (He), the observed Heterozygosity (Ho), the Polymorphic Information Content (PIC), Wright’s Inbreeding Coefficient (Fis) was calculated according to Wright (1931).

Furthermore, linkage disequilibrium and Hardy-Weinberg equilibrium (HW) tests were conducted using the same dataset. An AMOVA was employed to analyze the molecular variation among the groups under 999 simulations. By computing HW per locus per population, the nonrandom mating and population division hypothesis was obtained. A Minimum Spanning Network (MSN) was used to represent the genetic links between individuals and to measure them using Bruvo’s distance. A Discriminant Analysis of Principal Components (DAPC) was also used to determine the number of groups (clusters) and infer population structure (Jombart et al. 2010). The RStudio suite (V 1.2.5033; R V 3.6.2) and the Poppr (V. 2.8.5) package (Kamvar et al. 2015) were used to run all statistics and analyses.

Additionally, Structure 2.3.4 was used to determine genetic associations using a Bayesian approach (Pritchard et al. 2000). Ten independent repeats per K value (ranging from 1 to 10) were conducted using the admixture model. Each run included a burning phase with 100,000 iterations and a 500,000-iteration post-burning simulation.

## Results

The genotype accumulation curve showed that a plateau is reached using the first six loci, suggesting a great discriminating capacity providing robust genetic information across cow lineages when using the full 18 microsatellite genotyping panel. Across loci and populations, null alleles existed at less than 5% (mean of 0.19%) providing robust discrimination and statistics.

### Within breed diversity

A total of 18 microsatellite markers recommended by ISAG-FAO were used in this study to genotype Cyprus cattle. Allele sizes varied from 75 to 297 bp across loci. Polymorphism of the 18 microsatellite loci is presented in Table 2. A total of 168 alleles were detected at 18 microsatellite loci across the Cypriot breed. The total number of alleles (Na) ranged from a minimum of five alleles in ETH10 to a maximum of 16 alleles in TGLA53, with a mean of 9.33 ± 3.03 across loci. The Effective Number of alleles (Ae) varied from 2.65 (BM213) to 6.09 (TGLA53), with a mean of 3.98 ± 1.02 across loci. The expected heterozygosity (He) per locus fluctuated from a minimum of 0.62 (BM2123) to a maximum of 0.84 (TGLA53), with a mean of 0.74 ± 0.064 across loci. The mean observed heterozygosity (Ho) per locus varied from 0.586 (BM2112, ETH225) to 0.905 (TGLA53), with a mean of 0.691 ± 0.084 across loci. Wright’s Inbreeding coefficient (Fis) ranged from − 0.104 (TGLA122) to 0.294 (ETH225), with a mean of 0.066 ± 0.10 across loci. The Polymorphic Information Content (PIC) varied from 0.62 (BM2113) to 0.84 (TGLA53), with a mean of 0.73 ± 0.06 across loci. Only one marker showed significant deviation from the Hardy Weinberg Equilibrium (RM067).

**Table 2:** Polymorphism of 18 microsatellite loci across the indigenous breed of Cyprus bovine cattle

locus		Na	Ae	He	Ho	PIC	Fis	HW
TGLA227	(D18S1)	14	4.17	0.75	0.681	0.76	0.092	NS
BM2113	(D2S26)	7	2.65	0.62	0.586	0.62	0.055	NS
TGLA53	(D16S3)	16	6.09	0.84	0.905	0.84	-0.077	NS
ETH10	(D5S3)	5	3.83	0.74	0.716	0.74	0.032	NS
SPS115	(D15)	6	3.14	0.68	0.724	0.68	-0.065	NS
SPS113		9	4.87	0.80	0.784	0.79	0.020	NS
CSRM60	(D10S5)	12	4.68	0.79	0.733	0.79	0.072	NS
MGTG4B		13	5.23	0.81	0.629	0.81	0.223	NS
CSSM66	(D14S31)	10	3.93	0.75	0.716	0.75	0.045	NS
ILSTS006	(D7S8)	7	3.30	0.70	0.707	0.70	-0.010	NS
RM067		9	3.70	0.73	0.621	0.73	0.149	*
TGLA126	(D20S1)	6	4.22	0.77	0.724	0.76	0.060	NS
TGLA122	(D21S6)	9	3.40	0.71	0.784	0.71	-0.104	NS
INRA23	(D3S10)	8	3.67	0.73	0.707	0.73	0.032	NS
BM1818	(D23S17	7	3.31	0.70	0.603	0.70	0.139	NS
ETH3	(D19S2)	8	2.73	0.64	0.629	0.63	0.017	NS
ETH225	(D9S1)	12	5.83	0.83	0.586	0.83	0.294	NS
BM1824	(D1S34)	10	2.87	0.65	0.595	0.65	0.085	NS
mean		9.33	3.98	0.74	0.691	0.73	0.066	
st. deviation		3.029	1.02	0.064	0.084	0.06	0.100	

Locus (genetic locus), Na (number of alleles observed at each locus), Ae (Effective Number of Alleles), He (Expected Heterozygosity), Ho (Observed Heterozygosity), PIC (Polymorphic information content), Fis (Wright’s Inbreeding Coefficient), HW (Hardy Weinberg Equilibrium)

Genetic diversity indices of the Cyprus Bovine Breed across the four prefectures of Cyprus are shown in Table 3. The total population sampled was 116 animals, varying from 11 cattle from Larnaca to 48 cattle from Limassol. The average number of alleles (Na) ranged from 4.72 (Nicosia) to 8.06 (Limassol), while the effective number of alleles (Ae) ranged from 11 (Larnaca) to 46.1 (Limassol). The expected heterozygosity (He) fluctuated from 0.674 (Nicosia) to 0.739 (Limassol) and the observed heterozygosity (Ho) ranged from 0.667 (Larnaca) to 0.729 (Nicosia). The subpopulation fixation index (Fis) varied from − 0.0816 (Nicosia) to 0.0972 (Pafos). Significant HW equilibrium deviation was found only in Limassol.

**Table 3:** Genetic diversity of Cyprus bovine breed across four regions of Cyprus based on 18 microsatellite loci

Pop	*N*	Na	Ae	He	Ho	Fis	IC	HW
Limassol	48	8.06	46.1	0.739	0.718	0.0284	-0.132-0.188	*
Larnaca	11	4.89	11	0.723	0.667	0.0775	-0.0827-0.238	NS
Nicosia	16	4.72	14.2	0.674	0.729	-0.0816	-0.242-0.0785	NS
Pafos	41	7.39	41	0.720	0.650	0.0972	-0.0629-0.257	NS

Pop (Population), N (Number of Individuals), Na (average number of alleles), Ae (Effective number of alleles), He (Expected Heterozygosity), Ho (observed heterozygosity), Fis (Wright’s Inbreeding Coefficient), IC (Confidence interval), HW (Hardy Weinberg Equilibrium)

### Between breeds diversity

**Fig. 2:**
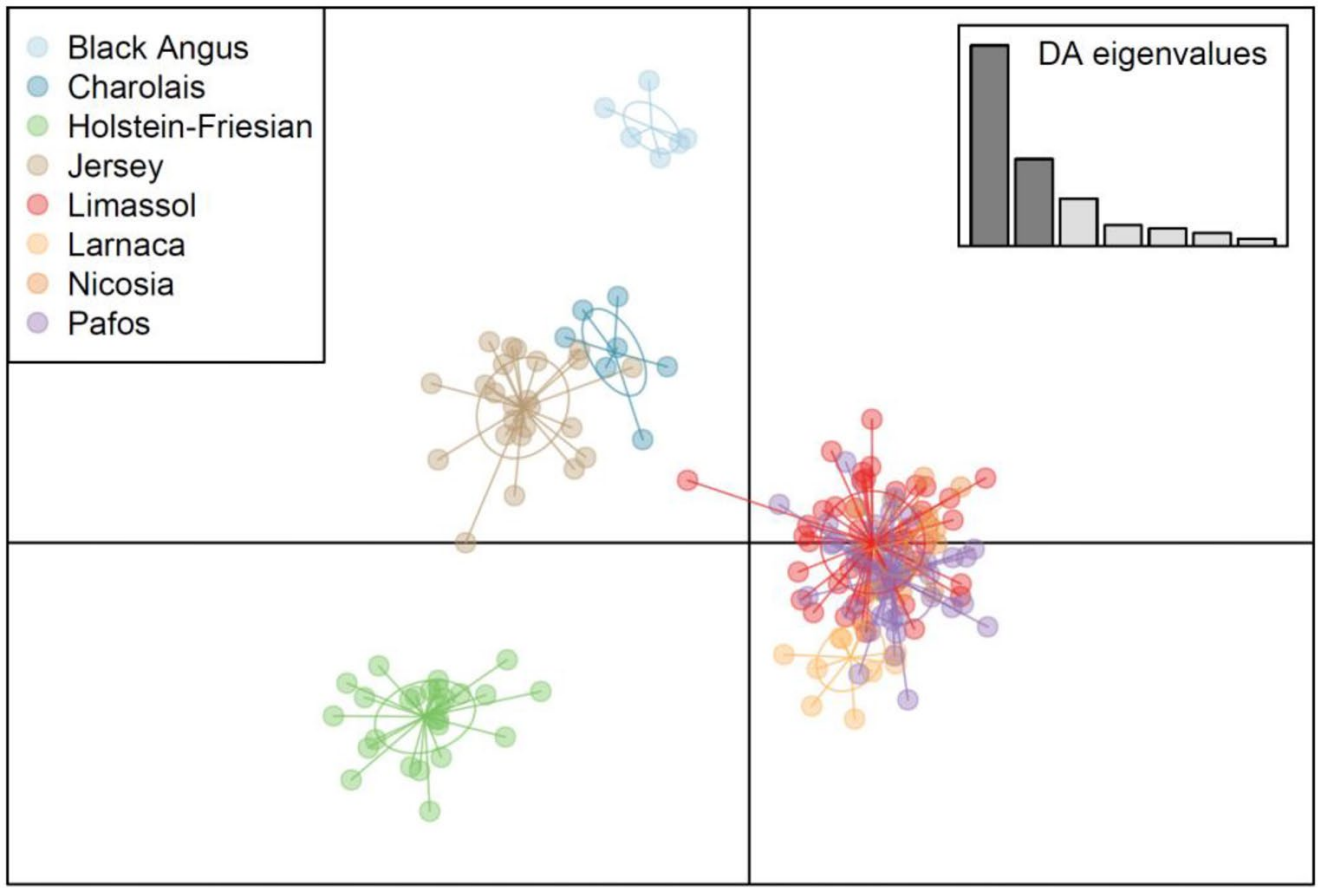
Discriminant analysis of principal components (DAPC) of Cyprus bovine breed, Blanck Angus, Charolais, Holstein-Friesian and Jersey breeds

In Fig. 2 the discriminant analysis of principal components (DAPC) was used to infer the number of clusters of genetically related individuals. A clear genetic separation of the Cypriot breed from the imported commercial breeds grown in Cyprus was established. The analysis of molecular variance showcased no significant differentiation of the population of Cyprus Bovine Breed between the different provinces.

**Fig. 3:**
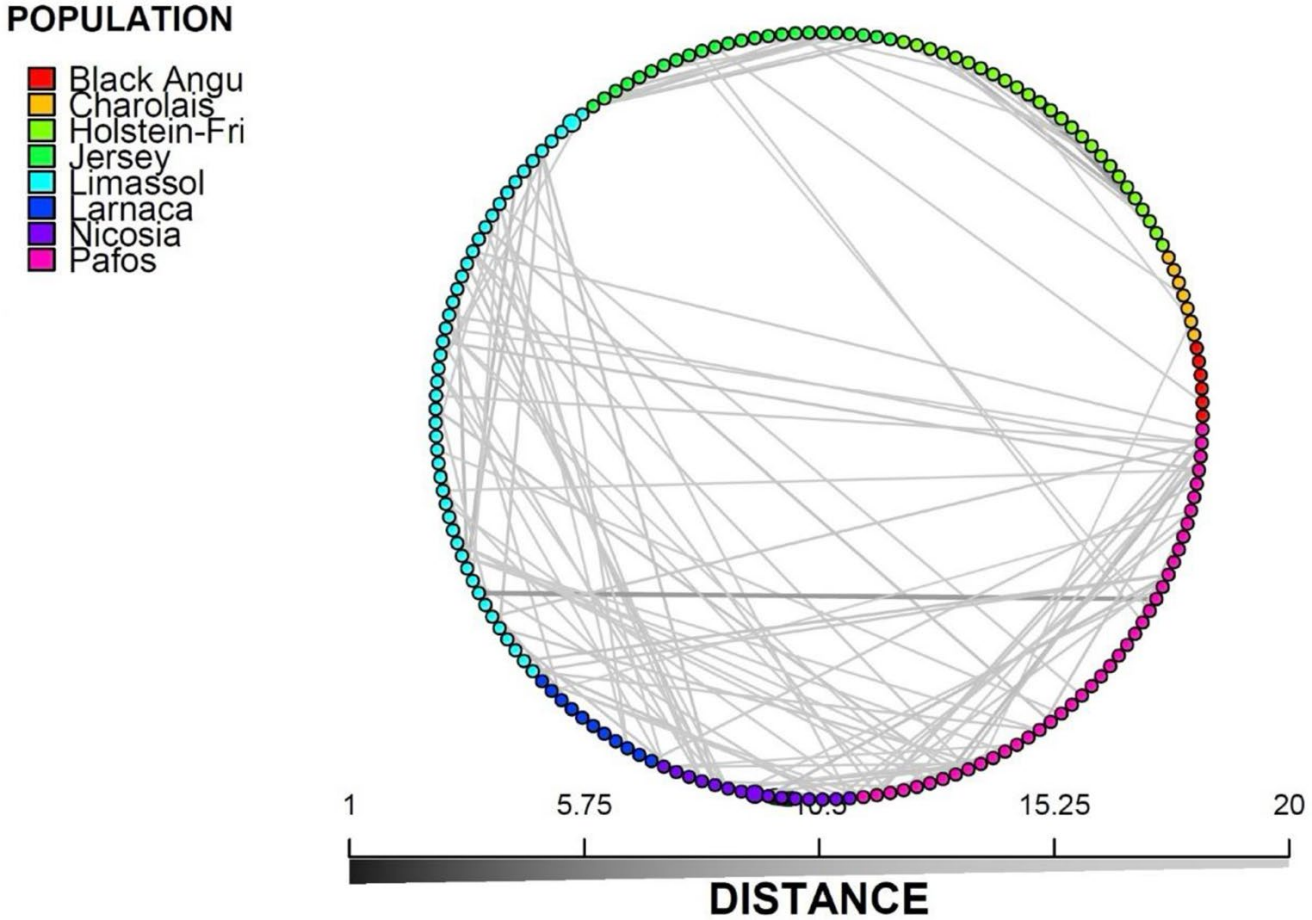
Minimum spanning network (MSN)

In addition, the minimum spanning network (MSN) analysis (Fig. 3) depicting the genetic affiliation across different breeds, provides evidence for breeding across Cypriot cows within and across prefectures, with minimum interconnections between the Cyprus Bovine cattle and any of the imported commercial breeds used in Cyprus. According to AMOVA analysis, nearly 93% of the variation is attributed within the population.

**Fig. 4:**
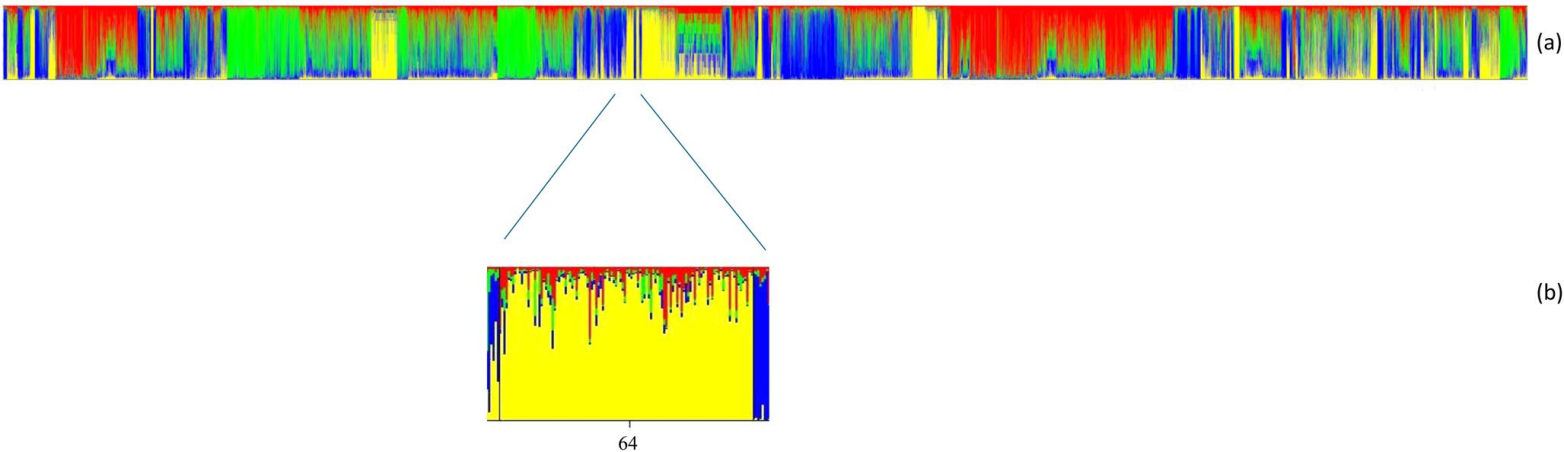
Structure analysis of each individual from the 163 analyzed cattle breeds and populations for K = 4, calculated from microsatellite data. Each individual is represented by a separate column, divided into K colours. Number 64 is the Cyprus Bovine breed. (**a**) Structure of 25,141individuals belonging to 163 populations. (**b**) Structure of 116 individuals belonging to the Cyprus Bovine breed

In Fig. 4, the structure analysis of 163 cattle breeds and populations is depicted. Bayesian analysis of population structure across breeds shows that the most probable classification is K = 4, with the Cyprus bovine breed exhibiting genetic affinity to Angola, Ankole Watusi, Bafata, Baladi, Brahman, Central African zebu, East African Shorthorn Zebu, Ethiopian zebu, Guzerat, Gyr, Indian Zebu, Kuri, Landim, Nelore, Pokot, Red Bororo (Zebu), Sindi, Sokoto Gudali (Zebu) and Sanga Tonga breeds. Further analysis of the clusters in the Cyprus Bovine Breed reveals that the second cluster (yellow) is the most dominant among all clusters. The first cluster (red) has a higher proportion in approximately 30% of the population compared to the third cluster (green), while the green cluster dominates over the red cluster in about 10% of the population. This variation in cluster proportions further highlights the differing degrees of genetic admixture within the Cyprus Bovine Breed.

## Discussion

The present study provides a comprehensive analysis of the genetic diversity and structure of the Cyprus Bovine Breed using 18 microsatellite markers recommended by ISAG-FAO. The results show the high discriminatory power of the selected markers and provide significant insights into the population structure and genetic diversity of the Cypriot cattle population, both within the breed and in relation to imported commercial breeds and other breeds throughout the world.

In this study, 116 individual cattle were analyzed, consisting of nearly 10% of the total Cyprus Bovine Breed population (Cypriot Ministry of Agriculture 2023). Blood samples from 63 individuals of imported commercial breeds used in animal farming in Cyprus were also sampled and compared to the Cyprus Cattle breed. Moreover, an online microsatellite database, consisting of approximately 25,000 individuals with over 160 breeds throughout the world genotyped by 30 microsatellites, was integrated into the data generated by this study (Solodneva et al. 2023).

The observed and expected heterozygosity values (Ho = 0.691, He = 0.74) indicate a moderate to high level of genetic diversity within the Cyprus Bovine Breed. This is further supported by the total number of alleles (Na) ranging from 5 to 16, with a mean of 9.33 across loci. This kind of genetic variability aligns with the higher end of the range typically reported for other cattle breeds worldwide, which generally varies from 7 to 9 (Cañón et al. 2001; Mateus et al. 2004; Lirón et al. 2006). The effective number of alleles (Ae = 3.98) and the polymorphic information content (PIC = 0.73) are comparable to those reported for other indigenous cattle breeds (Dalvit et al. 2008; Simčič, Horvat and Jovanovac 2008).

Despite the population being significantly small, considerable genetic variation is present across all prefectures, with Limassol and Nicosia showing higher observed heterozygosity (Ho). The lower Ho observed in Larnaca may be attributed to the very low number of farms growing the Cyprus cattle breed in that perfecture, thus limiting the sampling to two farms. The differences noted in genetic diversity could also be due to different reproductive management between the prefectures (Flores et al. 2023). This indication is further supported by the average number of alleles (Table 3).

These results reveal significant genetic diversity within the Cyprus Bovine breed, with no evidence of admixture with high-yielding imported commercial breeds. In contrast, the Burlina breed, while also demonstrating high genetic diversity, exhibited signs of crossbreeding with commercial breeds (Dalvit et al. 2008). Similarly, a study by Martínez et al. (2012) attributed the high genetic variability observed in Creole cattle, even in populations considered endangered, to the contributions of cattle from diverse origins that were recently admixed.

Additionally, the observed deviations from Hardy-Weinberg equilibrium were minimal, with only one marker (RM067) and only one prefecture (Limassol) showing significant deviation. This suggests that there is evidence of low or no selection within the Cyprus Bovine Cattle population, in agreement with Papachristou et al. (2020) referring that the breed is under weak artificial selection or not under any kind of coordinated artificial selection and in accordance with oral responses of the farmers to the questionnaires conducted in parallel with this study (data not shown).

According to Hartl (2000) positive magnitudes of the Fis, the values observed in the Cyprus Bovine Breed indicate a mostly low to average level of inbreeding within the population. The mean Fis of 0.066 across loci falls within the average range. The range of Fis values across loci, from − 0.104 (TGLA122) to 0.294 (ETH225), shows variability in inbreeding across specific loci, with several loci (TGLA53, SPS115, ILSTS006, TGLA122) exhibiting heterozygote excess. However, only one marker (MGTG4B) is within the range 0.16–0.25 and one marker (ETH225) exceeds the Fis level (> 0.25). Across prefectures, the Fis values ranged between − 0.0816 (Nicosia) to 0.0972 (Pafos), which indicates low to average inbreeding within the populations from different areas. Negative inbreeding suggests heterozygote excess, and the population is regarded as outbred.

According to Flori et al. (2019), the Fis value for the Cyprus Bovine breed was reported as 0.0259, corresponding to an average Fis value (Hartl 2000) aligns with the findings of the present study. In contrast, Papachristou et al. (2020) reported an inbreeding value of 0.261 for the Cyprus Bovine breed, which is considered high. This value may be attributed to the very small sample size of five individuals in the Papachristou study, which could have been sourced from a single farm or consisted of closely related animals, thus not accurately representing the broader population. The reduced genetic variation and the high inbreeding coefficient were attributed to the genetic drift, which is strengthened by geographical isolation, i.e., a breed on an island, that further prevents animal exchange (Papachristou et al. 2020). Since in the current study, 10% of the breed population was sampled, Fis estimations here must be regarded as representative of the indigenous Cyprus Cattle.

### Genetic diversity between the Cyprus bovine breed and other breeds

#### Population structure and genetic differentiation

The analysis of molecular variance (AMOVA) indicated that 93% of the genetic variation is found within individuals, while only a minor proportion (7%) being attributed to differences among populations across Cypriot prefectures, suggesting a high level of genetic connectivity within the Cyprus Bovine Breed, with little evidence for genetic differentiation between prefectures. Mamogobo et al. (2021)observed that most genetic differentiation, among indigenous breeds of South Africa, occurs within populations rather than between them. This was likely attributed to the absence of selection for specific production traits in such populations.

The discriminant analysis of principal components (DAPC) and minimum spanning network (MSN) further confirmed the close genetic affiliation among Cypriot cattle across prefectures, with no evident interbreeding between the Cyprus Bovine Breed and the imported commercial breeds, thus distinguishing the Cyprus Bovine Breed from commercial cattle breeds imported into the country. One Cyprus Bovine individual showed a genetic shift toward the Charolais and Jersey breeds. If that sample of the Cyprus cattle breed is removed, the 93% of the variation that is attributed within the population in the AMOVA analysis is expected to increase. However, this individual is still distantly related to the imported commercial breeds and the shifting could thus be possibly attributed to minimal interconnection of breeds in the past supported by the MSN.

DAPC positioned Charolais and Jersey close together. Since the vast majority of all farmed bovine individuals in Cyprus (over 96%) belong to Holstein Friesian, with only 3000 animals belonging to all other breeds (Cypriot Ministry of Agriculture 2023), crossbreeding of those few animals cannot be excluded. The DAPC also suggests that the originally found subtypes within the Cyprus Bovine breed, the “Mesaoria” type, also known as the plain areas type and the “Paphos” or the mountainous type, have being unified into one genetically similar group in accordance with data from producers exchanging males for reproductive purposes to avoid inbreeding. Indeed, the breed exhibited diversity among individuals, while still maintaining notable morphological, functional and genetic homogeneity.

#### Comparison with other cattle breeds

This study integrates diverse datasets spanning African, Creole, European, and Indicine cattle populations, offering a deeper understanding of the genetic influences shaping the Cyprus Bovine breed. The comparison of the Cyprus Bovine Breed with the online microsatellite database of 163 global cattle populations, revealed a close genetic affinity with several Bos indicus and African taurine breeds, including Angola, Ankole Watusi, Bafata, Baladi, Brahman, Central African zebu, East African Shorthorn Zebu, Ethiopian zebu, Guzerat, Gyr, Indian Zebu, Kuri, Landim, Nelore, Pokot, Red Bororo, Sindi, Sokoto Gudali and Sanga Tonga (Fig. 4). According to Martínez et al. (2012), Ginja et al. (2019) and Solodneva et al. (2023), Brahman, Guzerat, Gyr, Nelore and Sindi breeds were classified as Zebu cattle; while Angola, Ankole Watusi, Bafata, Baladi, East African Shorthorn zebu, Kuri, Landim, Pokot, Red Bororo, Sanga Tonga and Sokoto Gudali were classified as African taurine. The Cyprus breed appears to have a complex mosaic genome, consistent with historical records indicating shared ancestral origins with Zebu and African Bos taurus cattle (Flori et al. 2019; Papachristou et al. 2020), resulting from both historical and recent admixture events with neighboring and geographically distant breeds, likely due to ancient trade routes and cattle migrations (Beja-Pereira et al. 2003; Cymbron et al. 2005; Ajmone-Marsan and Garcia, José Fernando Lenstra 2010).

In agreement with the findings of the current study, Papachristou et al. (2020) concluded that the Cyprus bovine breed compared to other European cattle breeds, i.e. Kastellorizo, Agathonisi breeds, was closer to the root of the dendrogram and thus, closer to Gyr, the Bos indicus representative, while the Cyprus breed hold an intermediate position between Bos indicus and the remaining European cattle breeds, among the first dimension of multidimensional scaling. Individuals belonging to the Cyprus breed shifted from the European taurus side to the African taurus side toward the zebu apex thereby suggesting zebu admixture in these populations (Flori et al. 2019). This clustering reflects the known migration history of cattle breeds and Neolithic farmers, with Cyprus playing a pivotal role as a crossroad for the dispersion of both animals and people (Payne and Hodges 1997; Flori et al. 2019). Its geographical location in the core region of cattle domestication highlights its significance in the spread of domesticated species and human populations.

According to Beja-Pereira et al. (2003) cattle breeds of Iberian Peninsula revealed a high degree of genetic diversity and complex relationships, with Iberian cattle showing closer genetic ties to African B. Taurus alleles. Similarly, Cymbron et al. (2005) identified a clear correlation between genetic and geographic distances of cattle breeds from the Mediterranean and Northern Europe. Notably, cattle from Italy and Greece displayed Bos indicus alleles, while Portuguese breeds carried African Bos taurus markers, highlighting the genetic influences from different regions, further supporting the hypothesis of cattle dispersion among the mediteranean area.

## Conclusion

The results of this study have significant implications for the conservation and management of the Cyprus Bovine Breed. The genetic diversity and minimal differentiation among regions suggest that the breed has retained considerable genetic variability despite modern agricultural practices and the introduction of commercial high yielding cattle breeds. The clear genetic separation between the Cyprus Bovine Breed and imported commercial breeds highlights the importance of maintaining the genetic integrity of this indigenous population. Conservation programs should focus on preserving the unique genetic traits of the Cyprus Bovine Breed while ensuring sustainable breeding practices that avoid genetic admixture with non-native breeds.

Measures should be taken for the exchange of animals between the prefectures showing negative (Nicosia) or low Fis value (Limassol) with farms located in prefectures exhibiting higher values (Larnaca and Pafos) to reduce inbreeding. The current suggested male-to-female ratio is 1:20. It is recommended to either lower the current ratio or even maintain it at the current level. Additionally, atypical or non-standard animals based on slight colour differentiations should not be excluded from the breeding population, as white spots or irregular patterns seemed not to indicate crossbreeding with other breeds and may instead reflect natural genetic variation within the breed. SSR marker genotypes used in the current study could be a useful tool for the culling of individuals from the population and for the mating design of a prospective conservation program.

In conclusion, the Cyprus Bovine Breed exhibits substantial genetic diversity with strong genetic cohesion across regions of Cyprus. The 18 microsatellite markers used in this study provide robust discriminatory power, facilitating effective genetic characterization of the breed. The results highlight the need to continue monitoring the genetic health of the population and implement conservation strategies to safeguard its genetic heritage. The breed’s close relationship with other Bos indicus and African Bos taurus breeds offers valuable insights into its evolutionary history and resilience to environmental challenges.

## Data Availability

The datasets generated and analyzed during the current study are available from the corresponding author on reasonable request.
